# Exposure to Livestock Feces and Water Quality, Sanitation, and Hygiene (WASH) Conditions among Caregivers and Young Children: Formative Research in Rural Burkina Faso

**DOI:** 10.4269/ajtmh.18-0333

**Published:** 2019-01-28

**Authors:** Francis Ngure, Aulo Gelli, Elodie Becquey, Rasmané Ganaba, Derek Headey, Lieven Huybregts, Abdoulaye Pedehombga, Armande Sanou, Abdoulaye Traore, Florence Zongo, Amanda Zongrone

**Affiliations:** 1 Independent Research Consultant, International Food Policy Research Institute (IFPRI), Washington, District of Colombia and Cornell University, Ithaca, New York;; 2 International Food Policy Research Institute (IFPRI), Washington, District of Columbia;; 3 Agence de Formation, de Recherche et d’Expertise en Santé pour l’Afrique (AFRICSante), Bobo-Dioulasso, Burkina Faso

## Abstract

Livestock farming is common in low-income settings as a source of income and animal-sourced food. However, there is growing evidence of the harmful health effects of proximity of animals to infants and young children, especially through exposure to zoonotic pathogens. Poultry ownership is almost universal in rural Burkina Faso. Poultry feces are a significant risk factor for enteric diseases that are associated with child undernutrition. To investigate the extent of exposure to livestock feces among young children and caregivers, we conducted direct observations of 20 caregiver–child dyads for a total of 80 hours (4 hours per dyad) and recorded water quality, sanitation, and hygiene (WASH)-related behaviors. We also undertook in-depth interviews with these caregivers and focus group discussions with separate groups of men and women who were poultry farmers. Poultry and other livestock feces were visible in all 20 and 19 households, respectively, in both kitchen areas and in the household courtyards where children frequently sit or crawl. Direct soil ingestion by young children was observed in almost half of the households (45%). Poor handwashing practices were also common among caregivers and children. Although latrines were available in almost all households, child feces disposal practices were inadequate. This body of research suggests an urgent need to adapt conventional WASH and livestock interventions to reduce the exposure of infants and young children to livestock feces.

## INTRODUCTION

Poor water quality, sanitation, and hygiene (WASH) conditions cause recurrent childhood infections such as diarrheal infections, soil transmitted helminthes, and trachoma. Some studies have found that diarrheal infections could increase the susceptibility to pneumonia.^1–3^ Diarrhea and pneumonia are among the leading causes of mortality in children aged less than 5 years in low- and middle-income contexts.^4^ Poor WASH conditions are also associated with child undernutrition, primarily through enteric infections.^2,5^ Cohort studies link stunting, a chronic and resilient form of undernutrition, to repeated childhood infections such as diarrhea.^6^ Besides causing diarrhea, recurrent fecal pathogen exposure is thought to contribute to a subclinical condition, environmental enteric dysfunction (EED),^7^ characterized by chronic inflammation of the small intestinal lining, a permeable gut, and subsequent immune system stimulation, and malabsorption of nutrients.

Despite the gains made in improving WASH conditions in low-income contexts, poor domestic animal husbandry can present significant health risks to humans.^8,9^ There is growing evidence that proximity of domestic animals and their feces to young children serve as sources of zoonotic fecal pathogens that could counteract the health and nutritional gains from intake of animal source foods.^10,11^ Animal feces contamination is extensive and likely more prevalent than human feces in low-income rural, peri-urban, and urban contexts, where free-scavenging poultry and livestock husbandry practices are common.^10,12^ Animal feces are also an important source of contamination for water sources with fecal pathogens.^12^ Overnight corralling of poultry and/or livestock within the same housing structure as infants and young children was found to be associated with EED^13^ and stunting.^10,13^

Major fecal–oral routes of pathogen transmission have been well defined and summarized in a schematic representation known as the F-diagram.^14^ These routes are fluids, fingers, fields (floors, earth, and dirt), flies, fomites (utensils’, tables’, and seats’ surfaces), and food. In the past, researchers and WASH practitioners have mainly focused on human feces as the most important reservoir of pathogenic bacteria, and child feces in particular.^1^ However, domestic animal excreta are an important and underemphasized source of fecal contamination.^15–17^ Moreover, formative research suggests young children often directly ingest animal feces or contaminated dirt (geophagy), which have extremely high concentrations of bacteria.^16,17^ It has been hypothesized that ingestion of animal feces might also contribute to EED and ultimately child stunting, perhaps even through nonpathogenic bacteria.^17,18^ Ingestion of soil and animals feces is a pathway not disrupted by the traditional suite of WASH measures, such as use of toilets and improved water facilities.^16,17^ Although handwashing could minimize ingestion of fecal pathogens, it is poorly carried out and less common in many low-income settings.^15–17^ It is also unclear how effective these traditional WASH measures are in disrupting other pathways outlined in the F-diagram, linking animal feces to young children health and nutrition outcomes.

This growing body of research therefore points to a significant gap in the ability of conventional WASH interventions to prevent ingestion of animal feces by young children. Moreover, cognizance of the nutritional importance of animal-sourced foods (ASFs) has resulted in a wide range of nutrition-sensitive interventions including livestock components that could potentially increase the risk of ingestion of livestock feces. In light of this background, this formative research study was conducted to document health risks to young children and mothers in households targeted by a nutrition-sensitive poultry intervention in rural Burkina Faso (*Soutenir l’Exploitation Familiale pour Lancer l’Élevage des Volailles et Valoriser l’Économie Rurale* [SELEVER]).^19^

The main objective of this study was to assess the exposure of livestock feces and WASH conditions among caregivers and young children at household level in rural Burkina Faso.

## METHODS

### Study setting.

The study area included three villages purposively selected from the pool of villages where SELEVER activities were piloted in Balé, Kossi, and Boulkiemdé provinces in Burkina Faso. *Soutenir l’Exploitation Familiale pour Lancer l’Élevage des Volailles et Valoriser l’Économie Rurale* is a 5-year project implemented by Agribusiness Systems International in Ouagadougou, Burkina Faso in partnership with local non-governmental organizations (NGOs), private institutions, and governmental services. It is a poultry value chain program that leverages agriculture development strategies and nutrition to increase poultry production and improve the nutritional status of women and children in the Centre Ouest and Boucle de Mouhoun regions.

The villages were selected based on the following criteria: 1) villages from zones targeted for potential expansion but not yet covered by the program, 2) rural communities with access to a main poultry market where livestock rearing was common, and 3) villages with a mix of ethnicities.

Most of the households in these communities are smallholder farmers who practice poultry and livestock keeping as part of a mixed livelihood. A total of 20 households with children in three age groups (6–12 months, 12–24 months, and 24–36 months, with seven caregiver–child pairs in each age group) were purposively selected for the direct observations and semi-structured interviews. Ten of the 20 households had flocks of more than 20 chickens and/or other fowls.

### Study design.

This was an observational study that used a mixed methods approach to generate both quantitative and qualitative data using direct observations, in-depth interviews (IDIs), and focus group discussions (FGDs). The study took a total of 5 weeks (October–November 2016) to complete, including training enumerators and pilot testing research tools, recruiting and enrolling study subjects, and data collection.

### Data collection.

Using in-depth observation tools developed by Ngure et al.,^16^ quantitative and qualitative data were collected for identifying major pathways of fecal–oral microbial transmission among infants and children in the SELEVER study population. The approach included 1) direct observation of 20 caregiver–child dyads for 4-hour periods, including hourly hygiene spot checks, 2) a semi-structured questionnaire on WASH conditions in the 20 households, and 3) FGDs with separate groups of men and women. Each of these steps is described in the following paragraphs.

All enumerators were trained for 1 week in October 2016. This training included visits to two SELEVER-assisted communities to pilot test the research tools.

### Direct observation.

Direct observation of caregiver–child dyads, livestock, and WASH-related behaviors was carried out for all 20 households. The observations were conducted for 4 hours in each household, from 8.30 am to 12.30 pm, when the caregiver and the child were most active and going about their daily routine. Two well-trained research staff conducted the observations. One of them focused on the caregiver and child and the other conducted spot-check survey every hour. The second observer also supported the first one in observing caregiver’s routine activities, especially when the caregiver and child were separated.

Every event where the infant touched or mouthed an object was recorded on paper using a time stamp. The position of the child including crawling and touching the ground was also recorded. The frequency of putting an object in the mouth was recorded and each object was classified as visibly dirty or not. Mothers’ handwashing practices and toileting-related behaviors were also recorded. Handwashing-triggering opportunities were predefined, before conducting the research, as key events through which the mother’s or child’s hands potentially came into contact with soil and feces, for example, cleaning the baby’s bottom after defecation, and after sweeping, farm work, and latrine use. These opportunities included critical points of introducing fecal contamination in food, for example, before food preparation, eating, and feeding the child.

A second set of observations was undertaken in parallel, using a semi-structured questionnaire and checklist to determine the WASH environment within the same households. Water quality, sanitation, and hygiene attributes such as existence and evidence of use of a handwashing station and functional latrine were recorded. Spot checks were also conducted at hourly intervals during the observation period to record the number of roaming animals, the presence of fecal material in the immediate household courtyard, and the cleanliness of the mother’s and child’s hands.

### In-depth interviews.

Once the observation was completed, IDIs were conducted with the mothers/caregivers regarding childcare and WASH practices, including disposal of animal feces. On average, each IDI took about 15 minutes. A total of 19 interviews were conducted with mothers of children aged 6–36 months.

### Focus group discussions.

Four FGDs were undertaken with a total of 40 participants, that is, two separate groups of men and women in two villages. One village was selected per region. Thiou village was selected because it was the only village from the Centre Ouest region. Siby village was selected in the Boucle de Mouhun region as the village included both Mossi and Dioula populations and had a larger market than Siono.

These included 18 mothers and 22 fathers from poultry-producing households, with children aged 6–36 months. Each group included at least eight participants, half of them owned flocks of more than 20 chickens and/or other fowl. The FGDs were guided by trained facilitators and explored the following: 1) livestock husbandry practices and the risks associated with fecal–oral microbial transmission in young children, 2) WASH knowledge and practices in the community, and 3) the scope and feasibility of possible interventions. Each focus group took about 2 hours. Trained enumerators recorded all FGDs and interviews using digital handheld audio devices. Data collection was completed within 2 weeks after the training.

### Data management and analysis.

Once the data collection was completed, the data collectors and the field supervisor reviewed the data quality jointly; debriefings were held with the interviewers to identify key themes and provide a preliminary code list for the analysis of the FGD and interviews. Audio recordings were transcribed from local languages (Moore or Dioula) and translated into French and English. The field supervisor cross-checked the quality of recorded data transcription for consistency. Transcriptions were de-identified and submitted to the research team for coding and analysis. The preliminary code list was further developed before manual analysis by thematic code list groupings. Relevant codes were added as need arose and emerged from the data.

Summary statistics were calculated from the in-depth observation frequency data. Frequency of mouthing events was summarized for each potential object of microbial transmission. Similarly, frequencies for caregiver handwashing and child defecation events were also summarized.

### Ethical consideration.

Ethical clearance was obtained from IFPRI Institutional Review Board (IRB) in Washington, DC, and the National Ethics Committee of Burkina Faso (*Comite national d’éthique pour la Recherche en Sante* in Burkina Faso). After explaining the study’s objectives and procedures, enumerators obtained verbal informed consent from respondents. Informed consent was requested from each of the household heads before the interviews. All the discussion guides were written in French, and the enumerators spoke both French and the local language. Participation in the observations, IDIs, and focus groups was completely voluntary. Participants were free to withdraw at any time by informing the research staff.

## RESULTS

### Household and demographic characteristics.

In the study villages, consistent with other rural Burkinabe villages, household structures were organized in residential clusters of families (referred herein as enlarged households). The enlarged households consisted of a number of smaller or “restricted” households grouped into a compound around a courtyard.

Twenty caregiver–child dyads were observed for a total of 80 hours. Basic household characteristics in the study population are summarized in Table 1. All mothers were married, and only four of them (20%) had attained primary education. Most of the households (95%) owned a private latrine, except for one household that shared a neighbor’s latrine. Only one household (5%) had a handwashing station and soap. Thirteen households (65%) had access to running water, a borehole, or a protected well, whereas the remaining seven (35%) had access to an unprotected well. All households relied on a plastic jerry can or clay pot for storing water.

**Table 1:** Maternal and household characteristics (*n* = 20) in three communities in Balés, Kossi, and Boulkiemdé provinces in Burkina Faso

Characteristic		Mean (SD)
Number of households living within compounds		2.3 (2.2)
Number of children living in households (restricted)		3.8 (3.0)
Number of children living in compounds		4.9 (5.2)
		*n* (%)
Caregiver gender	Female	20 (100)
Level of education	Illiterate	16 (80)
	Primary	4 (20)
Marital status	Married	20 (100)
Latrine ownership	Own	19 (95)
	Neighbor’s	1 (5)
Latrine	Ventilated	8 (40)
	Full	0 (0)
Handwashing	Handwashing station	1 (5)
	Soap at handwashing station	1 (5)
Primary water sources	Communal tap water	4 (20)
	Borehole	4 (20)
	Protected well	5 (25)
	Unprotected well	7 (35)
	River	0 (0)
Drinking water storage	Jerri can	11 (55)
	Clay pot	9 (45)
Water-scooping container	Specific scooping cup	7 (35)
At point of use	Any cup/other*	13 (65)

* Other: any other type of container, for example, plastic jug and pot.

### General hygiene.

Some 25% of mothers and 60% of children had visibly dirty hands at the beginning of the 4-hour observation period (Table 2). At the beginning of the 4-hour observation period, animals were seen in the kitchen area in 17 of 20 households (85%) and poultry feces were seen on the kitchen floor area in all households. Poultry feces were also seen in all compounds. No human feces were observed in all the compounds. The kitchen yard was swept in only six households (30%). Chickens (mean *n* = 14, range 0–41) and guinea fowls (mean *n* = 3, range 0–32) were observed roaming freely in all compounds. Other livestock were found in the compound, including goats, pigs, and cattle, both corralled and free to roam. Such livestock feces were observed in almost all households (95%).

**Table 2:** General hygiene characteristics of household environment (*n* = 20) in three communities in Balés, Kossi, and Boulkiemdé provinces in Burkina Faso*

Characteristics	*n* (%)
Caregiver’s hands visibly dirty	5 (25)
Baby’s hands visibly dirty	12 (60)
Diapers or child’s bottom not clean	10 (50)
Status of dwelling	
Unwashed utensils	15 (75)
Uncovered utensils	16 (80)
Uncovered food	11 (55)
Dirt (specifically clay)†	19 (95)
Spill on floor (food or drink)	8 (40)
Poultry feces visible on kitchen floor	20 (100)
Animals in kitchen	17 (85)
Hygiene status of compound	
Kitchen yard swept	6 (30)
Area where child plays is swept	6 (30)
Poultry feces visible	20 (100)
Human feces visible	0 (0)
Other animal feces visible‡	19 (95)

* For all the hourly spot checks.

† One household was made of smooth concrete floor.

‡ Other animal feces include livestock (sheep, goats, cattle, and pigs) feces.

### Frequency of vector–mouth contact.

Objects identified as major fecal–oral vectors by frequency of mouthing were food, children’s hands or feet, water, food service utensils, and toys or play objects (Table 3). The objects that were mouthed were generally visibly dirty, with few exceptions. Mothers or siblings’ hands were not put into children’s mouths during the observations. Eleven children mouthed wood or plastic picked from the ground. Soil within the courtyard where chicken freely roam and defecate was ingested by nine (45%) children. Notably, the majority of food given to children (79%) came into contact with visibly dirty hands or utensils before ingestion.

**Table 3:** Potential sources of fecal–oral transmission for children (*n* = 20) in three communities in Balés, Kossi, and Boulkiemdé provinces in Burkina Faso

Potential vector	No. of children (%)	Mean episodes (SD)*	% Visibly dirty†
Food‡	19 (95)	7.05 (4.45)	79 (95/121)
Baby’s hands or feet	15 (75)	2.15 (2.37)	97 (29/30)
Baby’s cup and spoon	13 (65)	1.45 (1.36)	82 (18/22)
Fruits	3 (15)	0.2 (0.52)	75 (3/4)
Toys	7 (35)	0.95 (1.76)	100 (12/12)
Soil	9 (45)	1.25 (1.94)	100 (10/10)
Mother’s breasts	12 (60)	4.3 (4.84)	75 (63/84)
Water	6 (30)	0.35 (0.59)	57 (4/7)
Stone	1 (5)	0.05 (0.22)	100 (1/1)
Chicken feces	0 (0)	0 (0.00)	–
Wood	11 (55)	1.65 (1.87)	96 (22/23)
Plastic	11 (55)	1.55 (2.28)	95 (21/22)
Clothes	9 (45)	0.9 (1.25)	100 (7/7)
Pot, pan, and buckets	10 (50)	0.8 (1.01)	100 (6/6)
Other§	19 (95)	8.9 (6.62)	95 (59/62)

* Mean number of times each of the objects was put into the mouth for each of the index child over the 4-hour observation period.

† This column shows the percent of the number of times a visibly dirty object was put in the index child’s mouth. In parentheses are the number of times those episodes happened out of the total episodes for each potential vector.

‡ Visibly dirty food referred to any food that came into contact with bare soil, soiled hands, visibly dirty utensils, or murky water.

§ Others refer to any other object/ items not included in the list, for example, maize cobs.

### Livestock husbandry practices.

Livestock were generally housed outside these compounds, although small animals were free to roam within the compound during the day. Poultry rearing was common in these villages. Chickens roamed freely, scavenging for food, during the day and were housed in corrals within the main compounds during the night. Children and poultry were often found sharing the same spaces, closely interacting on a regular basis.

Primarily women were the main caregivers for infants and young children, as well as for poultry, throughout the day. In this context, there were frequent opportunities for children to be exposed to chicken feces as the chickens roamed freely in the compound. Women were aware that children ingest chicken feces and would sweep compounds to try to minimize this risk. However, the frequency of sweeping compounds was reported to vary considerably, with some households sweeping once or twice a day and others less than once a month, particularly during the main farming season when mothers were very busy. Although sweeping removes the bulk of fresh feces, it is also likely to spread drier feces into small less visible particles. During in-depth observation, chickens were also found pecking from plates that young children were being fed from, or from pots used to prepare meals.

### Handwashing and child feces disposal.

During the 80 hours of observation, mothers washed their hands 15 times of 61 triggering opportunities (25%) (Table 4). Soap was used in only three events. Mothers washed hands before eating at four of nine triggering opportunities for handwashing (44%) and before preparing food at four of 14 opportunities (29%). Handwashing after toileting (two of 13 opportunities, 15%) and after disposal of animal feces (0 of two opportunities) was low. Hands were air-dried on 12 of the 15 handwashing occasions. Drying with a clean cloth was not observed.

**Table 4:** Mothers’ handwashing (*n* = 20) in three communities in Balés, Kossi, and Boulkiemdé provinces in Burkina Faso

Event	Opportunities*	Any handwashing†	Handwashing with soap	Running water	Air-drying
After agriculture work	1	1 (100)	0 (0)	0 (0)	1 (100)
After cleaning animal feces	2	0 (0)	0 (0)	0 (0)	0 (0)
After playing on floor	1	1 (100)	0 (0)	0 (0)	0 (0)
After sweeping	7	1 (14)	1 (14)	0 (0)	1 (14)
After toilet	13	2 (15)	1 (8)	0 (0)	2 (15)
Before eating	9	4 (44)	0 (0)	0 (0)	3 (33)
Before feeding child	10	1 (10)	0 (0)	0 (0)	0 (0)
Before food preparation	14	4 (29)	0 (0)	0 (0)	4 (29)
Washing baby	1	1 (100)	1 (100)	0 (0)	1 (100)
Other‡	3	0 (0)	0 (0)	0 (0)	0 (0)

* Opportunities: these were key events when mothers’ hands were likely to come into contact with dirt and children, adult, or animal feces, as well as critical opportunities when dirt or fecal matter was likely to be introduced to/or contaminate food/or to be ingested during feeding.

† The same handwashing event could fit in all categories; for example, any handwashing could have been with soap and running water, and hands were air-dried.

‡ Other: any other opportunity of handwashing observed that was not classified under the rest of the categories.

During the 80 hours enumerators spent observing the 20 children, children’s hands were washed 15 times out of numerous and random handwashing opportunities, including after playing on the floor (six times), before eating (four times), and after eating (five times). Soap was used in only three of the 15 child handwashing events. Mothers washed their hands six of the 13 times when they cleaned their children after defecation, twice with soap. Of the 13 times children defecated, feces were disposed in latrines four times (31%) and in garbage pits three times (23%). Feces were thrown in the yard once. In four cases, feces were ignored for more than 30 minutes on the ground and their disposal was not observed. In one other case, the disposal method was not observed.

### Free-scavenging poultry and health risks.

Caregivers participating in the IDIs and FGDs gave advantages and risks of having children and chickens sharing the same space. The main perceived advantage given by the respondents was free roaming allowed chickens to scavenge for sufficient food both within and outside the courtyard to grow well and lay more eggs.

The main perceived disadvantage raised was the increasing risk of children ingesting chicken feces or contaminated soil, leading to illnesses such as diarrhea. During IDIs, one mother said,

Chickens and babies are together in the same compound. During the day, they spend a lot of time together since chicken peck where there are leftovers of food, and also poop there. So when they are alone, babies are at risk of eating chicken droppings, because they tend to put everything in their mouths, everything they touch.

A different mother said the following during the IDIs:

It’s difficult to monitor the children, with lack of attention children can eat soil, which contains the feces of poultry, goats. We certainly sweep the yard but all the dirt cannot be eliminated. We try our best to prevent chickens from having contact with children but that is difficult.

In addition, free-range chickens were in some cases stolen and had caused conflict between neighbors.

Most of the participants in the FGDs identified the opportunity to separate children from chickens by improving poultry housing, although cited “lack of means” as the main barrier to adapting such improvements. When asked about the need to separate poultry from children play areas one woman in the FGDs said,

Separating chickens from small children is the best way to prevent them from eating chicken droppings. This leads to illness, while we do not have the money to pay for prescriptions.

Two of the male participants in the FGDs said,

We would like to separate animals from humans in general. The current cohabitation is not interesting because the compound is never clean. The oxen, the goats, the sheep, the poultry remain in the compound at night, and during the day we do not always have pens. The ideal would be to build enclosures outside the compound for each type of animal.

For me, it is necessary to build a chicken house and to fence it for the big breeders. But those who do not have the means like us, we let chickens roam in the yard.

Several households in the study villages had been experimenting with local poultry-housing solutions that could also serve as models for other producers. These examples were cited as opportunities to improve the level of general household hygiene and also reduce the risk of children ingesting poultry feces.

### Water quality, sanitation, and hygiene knowledge and practices.

Although the concept of WASH was not widely understood, there was agreement among focus groups and IDI respondents on hygiene as it related to general cleanliness of the body, food preparation and storage (including utensils for cooking and eating), drinking water, and the household environment. In addition, participants agreed on the importance of latrine use, alongside handwashing with soap after latrine use, and of safe drinking water. These villages had benefited from previous small-scale WASH interventions, including latrine construction and water improvement. However, little or no hygiene-related sensitization activities had taken place.

## DISCUSSION

This mixed methods study presents a consistent picture of poor hygiene, particularly with regard to contamination of the immediate living environment with animal feces, poor child feces disposal, and handwashing practices. Poor animal husbandry practices present a pervasive health risk to vulnerable young children in the study area in rural Burkina Faso, especially in the context of poor general hygiene. Domestic animals were observed roaming freely in most of the households and chicken feces were observed in the kitchen areas and courtyards of all 20 households. Moreover, although all households reported access to a toilet, and human feces were not observed within children’s reach, poor disposal of children’s feces was common and presents an additional source of fecal contamination. Handwashing practices were also poor, and many mothers’ and children’s hands were visibly dirty. Respondents were generally aware of the health risks of poor hygiene, but this awareness did not translate into better hygiene practices.

Proximity and interaction of poultry and young children present health risks through exposure to fecal pathogens. Direct ingestion of soil within household courtyards, where poultry frequently scavenge and defecate, was observed among 45% of households. This is more frequent than what was observed in rural Zimbabwe (13%)^15^ and about the same as in Zambian households (47%)^15^ (albeit, in-depth observation periods were substantially longer in these other contexts). Direct soil ingestion by young children has also been observed in low-income contexts in rural Bangladesh,^20,21^ peri-urban Peru,^22^ and rural Kenya.^23^ In rural Bangladesh, 97% and 14% of the households (*n* = 216) exhibited detectable *Escherichia coli* and pathogenic *E. coli* in the soil, respectively.^20^ In the Peruvian study, *Campylobacter jejuni*, a pathogenic bacterium that causes dysenteric diarrhea, was isolated in 18% of chicken fecal samples 48 hours after deposition on a sun-exposed patio.^22^ These studies, and the present one, therefore suggest that soil ingestion is an important source of exposure to fecal pathogens for infants and young children.

This study also demonstrates how challenging it is for resource-constrained households to separate poultry from young children’s play and feeding areas. Corralling of poultry necessitates a package of interventions (improved feed and water supply, as well as vaccinations) that may be prohibitively costly for low-income households. Respondents cited these costs as the primary obstacle to separating animals from young children. Similar barriers to poultry corralling have been documented in a previous study in a Peruvian Shanty town^24^ and in formative research for the Sanitation Hygiene Infant Nutrition Efficacy (SHINE) Trial project in Zimbabwe.^16^ Whereas households in the Burkina Faso context prefer chicken to scavenge freely to get enough food for growth and eggs production, Peruvian households preferred free-range chicken for better tasting meat and eggs. It is worth noting that the caregivers in the IDIs and FGDs in rural Burkina Faso had good knowledge of health risks associated with free-scavenging chicken, especially the risk of diarrhea.

The FGDs also revealed knowledge of recommended WASH practices, but households in the study setting mostly failed to implement this knowledge. Only one quarter of all triggering opportunities resulted in handwashing by mothers, and use of soap was even more rare. Typically, parents did not wash hands after toilet use or disposal of animal feces, and mothers only washed their hands six of the 13 times they cleaned their children after defecation, and only twice with soap. Children’s hands were also visibly dirty most of the times they were put in their mouth. Handwashing for the 20 young children was observed 15 times during 80 hours of in-depth observation. However, given that young children frequently play, crawl, and feed from open courtyard soil, it is unclear how effective handwashing would be for young children in this kind of environment. Potentially, wet hands introduce further opportunities for young children to pick more contaminated soil while playing and crawling.^16^ The perception that such environment is normal for children to play and thrive in, coupled with poor handwashing knowledge and time constraints on the caregivers’ part, makes it harder to practice handwashing for children.

Poor hygiene practices are challenging to address and reflect multiple constraints. In rural Burkina Faso, water is implicitly costly because it requires collection from communal sources. Households are likely to ration water in response to these costs. Soap is also an additional cost, but potentially undervalued if households underappreciate the costs of poor hygiene practices. Time constraints are also likely to condition the capacity of caregivers to clean and sweep kitchen areas and the immediate household courtyards, although variation in these practices suggests some room for improvement. Improved handwashing practices generally require lowering the implicit cost of accessing water (e.g., tippy taps) and social behavior change communications interventions to raise awareness of the benefits of handwashing and of other hygienic practices.

For the young children, the problems are more complex because the fundamental problem is that children are often placed on unclean surfaces, often exposed to animals directly, and generally not closely supervised. The SHINE project in Zimbabwe promoted the use of a protective play space (washable play mat and play pen), which could protect children from contaminated soils. Another potential solution is volunteer-based day care centers where there are opportunities to create cleaner, animal-free spaces where children can be more closely supervised.^25^ Another set of solutions relies on more subtle behavioral changes, including closer supervision of children, improved management of household food waste, more frequent sweeping and cleaning of homestead floors, and safe management of child and animal feces. However, little is known about the effectiveness of these different interventions.

This study has some limitations. In-depth observation over prolonged periods necessitates a relatively small sample size, which limits the external validity of the analysis. Observations of child–caregiver dyads were limited to 4-hour periods at a specific time of year, resulting in 80 hours of observation. Although sufficient to observe frequent events, this may be insufficient to observe more infrequent events such as direct ingestion of animal feces. However, given the frequency of soil ingestion, it is very likely that animal feces in small invisible particles were frequently ingested.

Questions about WASH and childcare practices are also vulnerable to response biases—such as social desirability bias—especially if respondents are aware of the nature of the study. The use of mixed methods to study WASH and livestock husbandry practices was likely to minimize this type of bias. Hawthorne effects are also a concern for the observation of child–caregiver dyads because caregivers may alter their regular behaviors in response to being observed. Experience from in-depth observations in rural Zimbabwe, however, suggests mothers were comfortable and back to their usual routine after the first 1–2 hours of having the observers.

## CONCLUSION

Livestock husbandry, handwashing, and child feces disposal practices remain inadequate and provide immense opportunity for behavior change and technological approaches to reduce health risks among children and caregivers in the study context. Child-sensitive WASH practices are underemphasized in conventional WASH interventions, as are the problems of fecal contamination by livestock. Innovative interventions to address the gaps in conventional WASH could be critical to breaking fecal–oral pathways such as direct ingestion of contaminated soil.

More generally, issues surrounding children and livestock need to be integrated into more traditional WASH interventions. Young children are exceptionally vulnerable because of their weaker immune systems, their critical stage of cognitive and physical development, mouthing and other exploratory behaviors, and their dependence on caregivers. Behavior change approaches on sanitation should harness the missed opportunities of creating disgust and promoting improved management and disposal of animal feces in such an area in rural Burkina Faso and other similar contexts.

There is an important role for the agricultural sector to address these problems. The recent momentum in advocating for nutrition-sensitive agriculture, especially through promotion of ASFs, provides challenges and opportunities in this regard. Livestock programs not accompanied by WASH-sensitive livestock management practices are potentially harmful to young children. On the other hand, interventions that do encourage improved livestock corralling potentially yield an additional benefit. There are also opportunities for livestock extension agents to raise awareness of the risks of pathogen exposure from livestock feces and to promote a greater appreciation of the human health benefits from livestock corralling and hygiene.
